# A Home-Based Strength Training Intervention for Stress and Depression Symptoms in Obese Latino Adolescent Males—A Pilot Study

**DOI:** 10.3390/healthcare14050650

**Published:** 2026-03-04

**Authors:** Louise A. Kelly, Angela Martinez Dominguez, Muireann I. McMillan, Rory Petersen, Vanessa Facey, Miguelangel Bolio, Brooke Hugo

**Affiliations:** Health Disparities Research Laboratory, Exercise Science Department, California Lutheran University, Thousand Oaks, CA 91360, USA; amartinezdominguez@callutheran.edu (A.M.D.); muireann_mcmillan1@my.vcccd.edu (M.I.M.); rorypetersen@callutheran.edu (R.P.); vfacey@callutheran.edu (V.F.); mbolio@callutheran.edu (M.B.);

**Keywords:** adolescents, obesity, strength training, psychosocial health, salivary cortisol, pilot study

## Abstract

**Background:** Obese Latino adolescents are at increased risk for stress and depressive symptomology, but interventions to target both physiological and mental health outcomes are scarce. This pilot randomized controlled trial assessed feasibility and preliminary efficacy for a home-based strength training (HBST) intervention on stress-related and mental health outcomes in obese Latino adolescent males. **Methods:** Fifty-two adolescents were randomized to HBST (*n* = 26) or control conditions (n = 26). Participants randomized to HBST completed a 16-week progressive resistance training intervention performed twice per week at home on non-consecutive days. Primary and secondary outcomes were assessed at baseline and immediately post-intervention and included measures of upper- and lower-body strength (1RM), body mass index (BMI), BMI percentile, BMI Z-score, salivary cortisol, depressive symptoms (CES-D), and perceived stress (PSS-14). Results are presented using completer-only analyses (*n* = 25) and mixed-design ANOVA models. An ANCOVA sensitivity analysis was conducted for depressive symptoms due to baseline imbalance, including baseline CES-D as a covariate in the model. **Results:** Recruitment goals were met, but retention was lower than expected (48% overall; HBST = 31%, control = 54%). Analyses revealed a significant Time × Group interaction for salivary cortisol (F(1, 20) = 5.70, *p* = 0.027, ηp^2^ = 0.222), such that cortisol decreased over time in HBST participants and increased in control participants. While all strength and anthropometric outcomes improved descriptively from baseline to follow-up in the intervention condition, no significant interactions were present between groups. Depressive symptoms also decreased descriptively in HBST participants, but this effect was no longer significant after adjusting for baseline CES-D using ANCOVA (F(1, 19) = 0.002, *p* = 0.968). There were no significant findings for perceived stress. **Conclusions:** Differential effects were observed on salivary cortisol, suggesting HBST may be feasible in obese Latino adolescents. However, results should be interpreted with caution given baseline imbalance, small sample size, high attrition, and limitations with measuring cortisol at one time point without adjustment for time of day or key psychosocial and physiological confounders. All psychological and anthropometric outcomes were exploratory and non-significant after adjustment. A larger, multisite trial using baseline-adjusted analytic procedures, repeated physiological sampling, objective measures of adherence, and extended follow-up is needed to determine whether HBST produces meaningful effects that are sustained over time.

## 1. Introduction

Childhood and adolescent obesity are major public health issues in the United States, particularly for Latino youth, who face a disproportionately higher prevalence of obesity compared to their non-Latino peers [1,2]. Obesity during adolescence has been linked with negative cardiometabolic and mental health outcomes, including symptoms of stress and depression [3,4]. Mental health issues are particularly salient for adolescents since dysregulation can persist into adulthood and is associated with poor outcomes, including diminished academic achievement and social function and long-term cardiometabolic morbidity and mortality [5]. In fact, in this age group, symptoms of stress and depression are of particular concern since adolescence is marked by major physical and psychosocial changes that can impact youth in unique ways [5,6].

Latino adolescents have high levels of chronic psychosocial stress and are at an elevated risk for depressive symptoms yet are far less likely to access mental health services compared to their non-Latino White peers [6,7,8]. Stressors common to Latino youth often reflect environmental challenges that are not faced by adolescents of other ethnic backgrounds and go beyond typical normative stressors, including experiences of socioeconomic disadvantage, community violence, academic stress-related to first-generation college status, discrimination, and stress-related to immigration and acculturation-based pressures within the family [9,10,11]. Chronic or persistent activation of the physiological stress response to such psychosocial stressors has been linked to alterations in key biological systems such as the hypothalamic–pituitary–adrenal axis and dysregulation of neurobiological responses to stress [12]. In turn, such changes have been associated with increased vulnerability to depression and other negative mental health outcomes during adolescence [12].

Depressive symptoms in Latino youth are often overlooked and untreated, owing to a complex interplay of cultural, structural, and systemic barriers. Mental health stigma, low availability of culturally and linguistically responsive mental health care, language and cultural barriers, low insurance coverage, and transportation issues are among the key reasons why Latinos use mental health services at significantly lower rates than non-Latino White communities [8,13]. Latino adolescent boys may face additional barriers since prevailing social norms around masculinity that equate emotional expression with weakness and stress self-reliance may deter boys from revealing emotional struggles and seeking support from mental health professionals [14]. As a result, symptoms of stress and depression may be underreported by youth and caregivers and may manifest indirectly in the form of somatic symptoms, emotional and behavioral problems, and disengagement from school and physical activity, among others [15].

Obesity may also uniquely contribute to the experience of stress and symptoms of depression among Latino adolescents. Adolescents with obesity experience a greater prevalence of weight-related teasing, social isolation, and low self-esteem, all of which have been shown to be associated with depressive symptomatology in the general adolescent population [3,16]. Latino adolescents with obesity may face an intersection of stressors-related to their ethnicity, body weight, and social class that could exacerbate mental health risk [11,17]. Despite this, few mental health-focused interventions have been developed specifically for obese Latino adolescents, and to the best of our knowledge, none have focused on adolescent boys.

Culturally acceptable and accessible interventions that target multiple outcomes, both physical and mental health-related, would help alleviate unmet needs in this population. Physical activity is a low-cost and effective strategy for improving mental health and has been shown to reduce symptoms of stress and depression in youth populations [18]. Resistance training, in particular, has been found to improve symptoms of depression across a variety of clinical and non-clinical samples and may do so through mechanisms related to changes in self-efficacy, emotional control, body image, and underlying biological pathways involved in stress and mood regulation [12,19]. Resistance-based interventions may be especially relevant and attractive for adolescent boys, and their mental health benefits may be independent of aerobic fitness outcomes [19].

However, most of the literature has examined aerobic exercise interventions or predominantly female samples and included few adolescents from underserved ethnic groups [18]. Moreover, existing interventions tend to be supervised, facility-based programs that require travel and are often prohibitively costly, resulting in poor long-term adherence, particularly in Latino populations [8]. HBST programs may provide an alternative that is less burdensome from a financial and time-cost perspective, while also providing adolescents with greater independence and accessibility.

Multiple biological and psychosocial mechanisms may underlie the hypothesized relationship between resistance training and perceived stress and depressive symptomology. Dysregulation of the hypothalamic–pituitary–adrenal (HPA) axis has been noted as a result of chronic psychosocial stressors during adolescence, and disrupted cortisol secretion patterns have been linked with depressive symptom vulnerability and negative mental health outcomes [12]. Stress physiology and cortisol concentrations are decreased with exercise training protocols (including resistance exercise) [6,9,10], providing some evidence that exercise may serve as a regulator of HPA-axis activity [20]. Resistance exercise may also increase perceived competence via mastery experiences (e.g., regularly lifting weights) and observation of strength improvements. Adolescents are particularly sensitive to perceptions of competence due to developmental increases in perceived self-consciousness and body dissatisfaction [5]. Resistance training has also been noted to decrease depressive symptoms in clinical and non-clinical populations through mechanisms associated with improved emotion regulation, body image, and changes to depression-relevant biological pathways [12,19]. Strength-based exercise may be particularly beneficial and acceptable to obese Latino adolescent males who may be disproportionately affected by stress from weight stigma and discrimination and adherence to Latino masculine expectations [11,14,16]. Developmentally appropriate biological and psychosocial mechanisms provide rationale for the current investigation into home-based strength training to influence stress regulation and depressive symptoms in obese Latino adolescent males.

Resistance training has been shown to positively impact depressive symptoms across meta-analytic reviews and randomized trials [19]. However, much of this research has been performed in adult or mixed-age samples. Similar findings have been observed regarding participation in exercise leading to decreased stress and improvements in cortisol dysregulation among youth. These studies, however, have not always focused on resistance-based training nor been conducted solely among adolescent samples [6,9,10,20]. Furthermore, research specifically among obese Latino adolescent males is limited. Because of unique developmental, biological, and sociocultural influences faced by Latino adolescents that contribute to increased risk for psychosocial stress exposure and decreased use of mental health services [6,7,8,14], there is a need to conduct controlled trials among obese Latino adolescents to determine if resistance-based interventions impact physiological and psychological outcomes.

While the potential benefits of HBST in reducing stress and symptoms of depression are promising, especially for adolescents, relatively few studies have examined the mental health effects of structured HBST programs in obese adolescents, and even fewer have been performed in Latino youth, especially adolescent boys. This gap in the literature is a critical one to address given the aforementioned mental health disparities among Latino adolescents with obesity. HBST programs also have the potential to reach adolescents outside of the traditional school or clinical settings, further increasing access in underserved communities, and may provide an important tool for promoting physical and mental health in this population. Thus, the aim of the current study was to investigate the effects of a 16-week, twice-weekly, home-based, unsupervised strength training program on symptoms of stress and depression in obese Latino adolescent boys.

## 2. Materials and Methods

### 2.1. Study Design and Randomization

After completion of all baseline tests (outpatient and inpatient), subjects were randomly assigned to either the Home-Based Strength Training (HBST) group or the Control (C) group. Randomization was completed using a computer-generated random number sequence. Allocation was concealed from the participants and all study personnel until after the completion of all baseline testing. Post-intervention testing was completed 48–72 h after the last strength training session.

### 2.2. Feasibility Outcomes

Feasibility outcomes were incorporated as a pilot trial. Feasibility outcomes included recruitment, retention, and completion of follow-up assessment. Feasibility of recruitment was measured by how many eligible participants were recruited during the allotted recruitment timeframe. Feasibility of retention was measured by the number of participants who completed post-intervention assessments. Reasons for participant attrition were collected when available. The current work is a secondary analysis of a pilot trial, methods for which have been published previously [21]. Sample size calculations for differences in adiposity and risk factors for type 2 diabetes between intervention and control arms were performed for the parent trial. Parent trial power calculations were based on approximately 80% power, α = 0.05, and suggested that between 11 and 12 participants/group would be needed to observe differences in fat mass and insulin sensitivity primary outcomes.

The parent trial was powered for metabolic outcomes but did not have feasibility success parameters outlined a priori for recruitment, retention, and adherence specific to the behavioral intervention. To allow for maximum transparency related to the methods used to conduct this pilot behavioral intervention, we defined a priori feasibility parameters consistent with recommendations put forth for pilot and feasibility trials. Adequate feasibility for recruitment was considered to be the successful enrollment of ≥80% of eligible individuals approached during the recruitment period. Adequate feasibility for retention was defined as ≥60% of randomized participants completing follow-up assessments after intervention conclusion. Adequate feasibility for adherence was considered ≥70% of prescribed sessions completed as recorded by session attendance training logs.

### 2.3. Participants

Fifty-two participants (N = 52) were recruited from the greater Los Angeles County area through medical clinics, community outreach, and local schools to participate in the Families United for Education and Research for Strong Adolescent Latinos (FUERSA) study. Recruitment occurred over an 18-month period through community outreach, medical clinics, and local schools in the greater Los Angeles County area. Inclusion criteria included the following: (1) male; (2) in 9th–12th grade (approximately 14–18 years of age); (3) body mass index (BMI) percentile ≥ 95th for age and sex; (4) Latino ancestry, defined by parental and grandparental descent by self-report; (5) no diagnosed metabolic disease; and (6) not taking medication for stress or depression.

The study was performed according to the Declaration of Helsinki. Prior to participation, written informed parental consent and adolescent assent were obtained. The study was approved by the Institutional Review Board of the University of Southern California.

### 2.4. Anthropometric and Obesity Measurements

Height was measured to the nearest 0.1 cm using a beam medical scale and wall-mounted stadiometer (SECA, Hamburg, Germany), and body mass was measured without shoes and in a hospital gown to the nearest 0.05 kg using a beam medical scale. Body mass index (BMI) was calculated as weight in kilograms divided by height in meters squared (kg/m^2^). Age- and sex-specific BMI percentiles and BMI Z-scores were calculated using Epi Info™ (Version 7; Centers for Disease Control and Prevention [CDC], Atlanta, GA, USA) using Centers for Disease Control growth reference data. Children with a BMI ≥ 95th percentile were classified as obese. BMI Z-score represents the number of standard deviations from the mean, based on age- and sex-specific references, and was used as a continuous variable in analysis.

### 2.5. Strength Assessment

Upper- and lower-body muscular strength was measured as maximal strength using one-repetition maximum (1-RM) bench press [upper-body strength] and leg press (lower-body strength) protocols as described previously [22]. After undergoing a standard warm-up consisting of light aerobic activity and warm-up sets with lighter resistance on each exercise, subjects completed a series of single repetitions with progressively heavier loads to volition until 1 RM for each lift was determined. Resistance was increased incrementally until the subject was unable to lift the weight once with proper technique. Subjects were given sufficient rest between trials (2–3 min) to prevent fatigue. All trials were supervised by trained professionals. Test-retest reliability and validity of these measures have been demonstrated previously [22]. Maximal weight lifted on each lift (1-RM, kg) was used as the outcome measure for upper- and lower-body strength, respectively. These tests were completed at baseline and follow-up. Upper- and lower-body 1RM were used as secondary physical outcome measures to assess changes in muscular strength due to the intervention. Additionally, as measures of functional improvement, the 1RM assessments were used as manipulation checks to verify physiological receptivity to the HBST program and objectively measure if participants increased strength over the course of the 16-week intervention.

### 2.6. Stress Salivary Cortisol

Salivary cortisol concentration is considered to be a valid marker of circulating free (unbound) plasma cortisol and biologically active cortisol. At baseline, all participants collected a sample of salivary cortisol using the Salivette^®^ saliva collection system (Sarstedt, Newton, NC, USA). A dry cotton swab was placed in the mouth for 2 min, producing about 1 mL of passively absorbed saliva. Samples were stored at room temperature for 1–2 h before being taken to the laboratory the same evening and kept overnight at 4 °C. The following morning, samples were centrifuged for 10 min at 2500 rpm, and the saliva supernatant was then frozen at −70 °C until assayed.

Salivary cortisol concentrations were measured by an automated enzyme immunoassay (Tosoh AIA-600 II analyzer; Tosoh Bioscience, South San Francisco, CA, USA) at the General Clinical Research Center Core Laboratory. The sensitivity of the assay was 0.02 μg/dL. Inter- and intra-assay coefficients of variation were 7.8% and 3.4%, respectively [20].

### 2.7. Depression Scale (CES-D)

Depressive symptoms were measured using the Center for Epidemiologic Studies Depression Scale (CES-D). The CES-D is a self-report scale that measures symptoms commonly associated with depression and has been used in prior published work [23]. The CES-D is a 20-item questionnaire that asks individuals how often they experienced depressive symptoms during the past two-week period. Response options for each item are based on a 4-point Likert scale ranging from 0 (Rarely or none of the time) to 3 (Most or all of the time). Items are summed to create a total score, with higher scores indicating more depressive symptoms. Following convention, we used a total CES-D score of 16 or above to denote clinically elevated depressive symptoms. Previous work has demonstrated good internal consistency of the CES-D, with previously published Cronbach’s α values ranging from 0.85 to 0.90 in both adolescent and adult samples.

### 2.8. The Perceived Stress Scale (PSS-14)

Self-reported perceived stress was measured using the Perceived Stress Scale (PSS-14). The PSS-14 asks individuals to rate the degree to which situations in their life are unpredictable, uncontrollable, and overwhelming. Perceived stress was measured with the 14-item self-report PSS instrument that measures how much stress participants have perceived in the previous month. Scores on each item are based on a 5-point Likert scale with responses ranging from 0 (never) to 4 (very often). Total scores are computed by summing item responses, with higher scores indicating higher perceived stress. While there are no established clinical cutoff scores for the PSS-14, higher scores have been linked to negative physical and psychological health outcomes, including depression [24]. Internal consistency has been good for the PSS-14, and previously published Cronbach’s α values typically range from 0.78 to 0.91 in different populations.

### 2.9. Description of Intervention

The HBST intervention was described in detail elsewhere, but the main components are briefly outlined [21]. It was a 16-week progressive resistance training program, and it was periodized into three phases. The frequency of the training was twice a week on non-consecutive days, and the total duration of the sessions did not exceed 60 min. The exercises that were used for training could be performed at home with limited equipment. Those randomized to the HBST group were given training logs in which to record each training session they completed along with whether they completed the prescribed exercises and the length of the session. Logs were collected at check-ups by study staff and used as an adherence measure to the intervention. As the intervention was completed in participants’ homes unsupervised, adherence was only based on self-report, and therefore exercise fidelity could not be objectively confirmed. The individuals in the control group were asked not to take part in any other structured or research-based physical activity or exercise programs.

### 2.10. Statistical Analysis

Descriptive statistics are presented as means and standard deviations (M ± SD). Baseline-to-follow-up differences for each outcome variable were evaluated separately using 2 × 2 mixed-design analyses of variance (ANOVAs). Time (baseline vs. follow-up; within-subjects factor) and Group (intervention vs. control; between-subjects factor) were entered as independent variables. Variables included strength outcomes (1RM) for the upper and lower body, body mass index (BMI; kg/m^2^), BMI percentile, BMI Z-score, salivary cortisol, depressive symptoms (CES-D), and perceived stress (PSS). Strength assessments (1RM) were included as secondary physical outcomes to verify muscular adaptations and intervention compliance, while salivary cortisol and psychological measures were analyzed as primary outcomes of interest for the current study.

Normality and homogeneity of variance were assessed with Levene’s tests and equality of covariance matrices with Box’s M tests. Sphericity was assumed for all analyses because there were only two repeated measurement time points. Mixed-design ANOVA was used despite significant results from Box’s M because mixed-design ANOVA has been shown to be robust to violations of equality of covariance when there are two repeated measures [25]. Analyses were first conducted to examine Time × Group interactions to determine if changes between time points differed between groups. Analyses of main effects for Time and Group were also conducted. Partial eta squared () values are reported as measures of effect size and were interpreted as small (≈0.01), medium (≈0.06), and large (≥0.14) [26]. When appropriate, estimated marginal means are reported with Bonferroni-adjusted pairwise comparisons.

Due to the preliminary nature of this pilot investigation and resulting small sample size, greater emphasis was placed on the magnitude and direction of effect sizes and observed changes rather than *p*-values alone. This analytic approach was chosen based on published methodological recommendations for interpreting results from studies that include repeated-measures data [27,28,29]. All statistical procedures were performed using IBM SPSS Statistics (Version 30; IBM Corp., Armonk, NY, USA).

## 3. Results

### 3.1. Feasibility and Attrition

**Figure 1:**
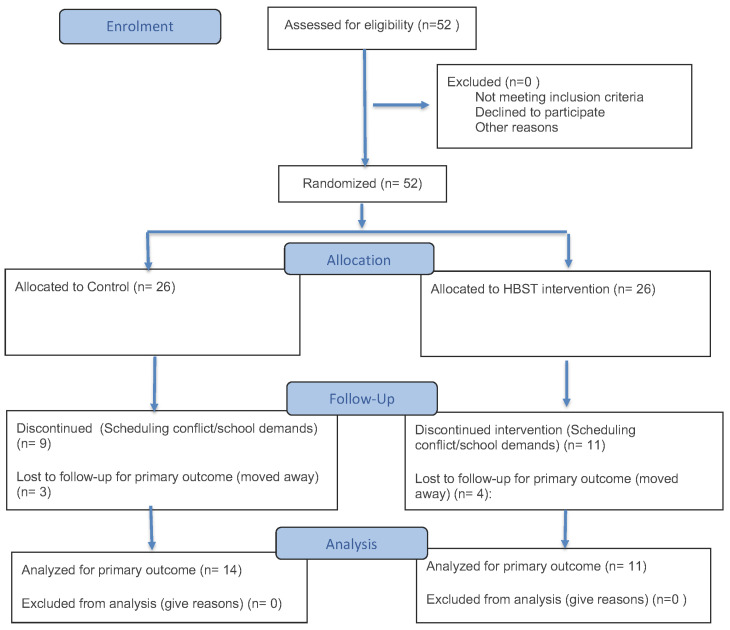
CONSORT flow diagram illustrating participant recruitment, randomization, attrition, and final analytic sample for the home-based strength training (HBST) intervention trial.

A total of 52 participants were recruited and randomized to either the HBST group (n = 26) or the control group (n = 26). Of these, 25 participants (48%) completed both baseline and follow-up assessments and were included in the final analyses (HBST: n = 11; Control: n = 14). Intervention completion was assessed among HBST completers using self-reported logs of home-based skill-training sessions. Although most intervention completers self-reported the number of sessions they completed, session completion was not objectively verified. Retention rates varied across conditions; 31% of HBST subjects and 54% of control subjects completed follow-up assessments. Common reasons for study attrition included scheduling conflicts, loss to follow-up, or conflict with academic and/or family commitments. No negative experiences with study participation were reported. A CONSORT flow diagram illustrating participant flow through each stage of the study (recruitment, randomization, attrition, analytic sample) is provided in Figure 1.

Recruitment goals were met within the allotted time period, suggesting that initial acceptability of the intervention was supported. However, retention was lower than expected, especially in the HBST condition; follow-up sessions may have been impacted by adherence to a lengthy (16 weeks) unsupervised home-based program. Results from feasibility were mixed; recruitment was successful, but retention was poor.

### 3.2. Anthropometric and Obesity Measurements

Of the 52 participants recruited, 25 completed both baseline and follow-up assessments and were included in the final analyses (the CONSORT Diagram can be seen in Figure 1). A similar pattern was present for all anthropometric variables. For BMI (kg/m^2^), a small reduction was observed between baseline (M = 32.83, SD = 4.57) and follow-up assessments (M = 31.86, SD = 4.51) in the intervention group, while participants in the control group experienced a small increase over time (baseline: M = 32.06, SD = 3.08; follow-up: M = 32.36, SD = 3.28) (see Table 1). BMI percentile also reduced from baseline (M = 98.10, SD = 1.56) to follow-up (M = 97.47, SD = 1.93) in the intervention group (see Table 1), while there was a small increase over time in the control group (baseline: M = 98.20, SD = 1.72; follow-up: M = 98.41, SD = 1.92). An analogous crossover pattern was present for Z-scores. Z-scores decreased between baseline (M = 2.23, SD = 0.41) and follow-up (M = 2.07, SD = 0.42) assessments in the intervention group (see Table 1). However, Z-scores increased between baseline and follow-up in the control group (baseline: M = 2.21, SD = 0.32; follow-up: M = 2.32, SD = 0.39). Estimated marginal means and profile plots demonstrate these divergent directions of change for each anthropometric variable. BMI (kg/m^2^). Inferential statistics revealed no significant Time × Group interaction, F(1, 21) = 2.45, *p* = 0.132, partial η^2^ = 0.104. There was no significant main effect of Time, F(1, 21) = 0.69, *p* = 0.416, partial η^2^ = 0.032. Additionally, there was no significant main effect of Group, F(1, 21) = 0.01, *p* = 0.930, partial η^2^ < 0.001. BMI percentile. Inferential statistics revealed no significant Time × Group interaction, F(1, 16) = 2.01, *p* = 0.176, partial η^2^ = 0.111. There was no significant main effect of Time, F(1, 16) = 0.50, *p* = 0.488, partial η^2^ = 0.030. Additionally, there was no significant main effect of Group, F(1, 16) = 0.40, *p* = 0.534, partial η^2^ = 0.025, Z-scores. Inferential statistics trended towards a significant Time × Group interaction, F(1, 16) = 4.23, *p* = 0.056, partial η^2^ = 0.209, which was a large effect. There was no significant main effect of Time, F(1, 16) = 0.14, *p* = 0.715, partial η^2^ = 0.009. Additionally, there was no significant main effect of Group, F(1, 16) = 0.44, *p* = 0.516, partial η^2^ = 0.027.

As many variables were not normally distributed, baseline differences between groups were assessed using Mann–Whitney U tests. There were no significant differences between groups on baseline CES-D scores (U = 52.00, z = −1.66, exact *p* = 0.103); however, the effect size was moderate (r = 0.32). As such, the CES-D score may still be unbalanced between groups. Participants assigned to HBST had significantly higher baseline salivary cortisol than controls (U = 29.00, z = −2.30, exact *p* = 0.021), with a moderate-to-large effect size (r = 0.47). Baseline differences did not reach significance on any other demographic or anthropometric variable (all *p* > 0.05).

### 3.3. Strength Assessment

Arm 1RM appears to have improved from baseline to follow-up in the intervention group (M change = +15.62, baseline M = 184.38, SD = 60.68, follow-up M = 200.00, SD = 71.91) but worsened over the same time period for participants in the control group (M change = −8.22, baseline M = 181.79, SD = 58.56, follow-up M = 173.57, SD = 75.94) (See Table 2). However, there is high variability in scores within each group. The inferential test showed that there was not a significant Time × Group interaction, F(1, 20) = 0.50, *p* = 0.486, partial η^2^ = 0.025. These results suggest that there were no significant differences between groups in how upper-body strength changed over time. There was also not a significant main effect of Time, F(1, 20) = 0.05, *p* = 0.828, partial η^2^ = 0.002, and not a significant main effect of Group, F(1, 20) = 0.35, *p* = 0.563, partial η^2^ = 0.017. *Leg 1RM:* Similar to Arm 1RM, descriptive statistics show opposing trends in scores between groups. Leg 1RM scores appear to have increased from baseline to follow-up in the intervention group (M change = +96.11, baseline M = 491.11, SD = 167.07, follow-up M = 587.22, SD = 264.61) (see Table 2) but decreased over the same time period for participants in the control group (M change = −68.21, baseline M = 522.50, SD = 144.92, follow-up M = 454.29, SD = 146.81). The inferential test revealed no statistically significant Time × Group interaction, F(1, 21) = 3.67, *p* = 0.069, partial η^2^ = 0.149. The interaction did not meet the conventional threshold for statistical significance (α = 0.05). In particular, the effect size can be interpreted as a moderate difference in how lower-body strength changed between groups from baseline to follow-up. There was not a significant main effect of Time, F(1, 21) = 0.11, *p* = 0.748, partial η^2^ = 0.005, and not a significant main effect of Group, F (1, 21) = 0.65, *p* = 0.429, partial η^2^ = 0.030.

### 3.4. Stress Salivary Cortisol

Descriptively, there was a crossover pattern in cortisol between groups. Cortisol levels decreased over time in the intervention group from baseline (M = 14.93, SD = 4.28) to follow-up (M = 12.23, SD = 2.46). Cortisol levels increased from baseline (M = 9.99, SD = 3.47) to follow-up (M = 12.21, SD = 4.28) in the control group. Inferential statistics showed a significant Time × Group interaction, F(1, 20) = 5.70, *p* = 0.027, partial η^2^ = 0.222, a large effect size. This suggests that the difference in cortisol levels between baseline and follow-up was significantly different between groups, such that the intervention group had reductions in cortisol compared to controls. There was a non-significant main effect of Time, F(1, 20) = 0.05, *p* = 0.819, partial η^2^ = 0.003, suggesting that cortisol did not significantly increase or decrease from baseline to follow-up when considering both groups together. There was a marginally significant main effect of Group, F(1, 20) = 3.57, *p* = 0.073, partial η^2^ = 0.151. The significant Time × Group interaction and associated effect size indicate that changes in cortisol differed between groups over time.

### 3.5. Depression CES-D

The HBST group had higher depressive symptom scores (CES-D) at baseline compared with controls (Table 1). Because of this difference at baseline, when CES-D scores were averaged over time points, there was a significant main effect of group. Descriptively, CES-D scores decreased from pre-test (M = 20.75, SD = 9.15) to post-test (M = 17.13, SD = 8.11) in the intervention group. Conversely, depressive symptoms increased from pre-test (M = 13.50, SD = 3.98) to post-test (M = 14.50, SD = 5.65) in the control group. Estimated marginal means plots show profile plots consistent with this pattern, with depressive symptoms decreasing from pre-test to post-test in the intervention group and increasing over time in the control group. However, inferentially, there was no significant Time × Group interaction, F(1, 20) = 1.94, *p* = 0.179, partial η^2^ = 0.088. There was also no significant main effect of Time, F(1, 20) = 0.62, *p* = 0.439, partial η^2^ = 0.030. There was a significant main effect of Group, F(1, 20) = 4.49, *p* = 0.047, partial η^2^ = 0.183, indicating greater depressive symptoms in the intervention group when averaging across time points. Follow-up post hoc comparisons using estimated marginal means showed that CES-D scores were significantly greater in the intervention group compared to the control group, *p* = 0.047. Although depressive symptoms did not significantly decrease from pre-test to post-test more in the intervention group compared to the control group, this may have been due to low power, as there was a moderate-to-large effect size for this comparison and CES-D scores decreased in the intervention group. As noted above, there was a baseline imbalance in depressive symptoms. To ensure that this did not influence our results, we repeated our ANCOVA using follow-up CES-D as the dependent variable, with baseline CES-D entered as a covariate. Results showed that after controlling for baseline levels of depressive symptoms, group did not have a significant effect on follow-up CES-D, F(1, 19) = 0.002, *p* = 0.968, partial η^2^ = 0.000. This suggests that any between-group differences observed in depressive symptoms at follow-up were largely due to baseline imbalance.

### 3.6. Perceived Stress Scale (PSS)

On a descriptive level, PSS scores from baseline to follow-up showed a slight increase for the intervention group (from M = 20.38 to M = 20.88, SD = 1.60 to SD = 4.29), whereas perceived stress levels decreased slightly among the control group during this same time period (baseline M = 21.38, SD = 2.99; follow-up M = 20.77, SD = 6.06). Estimated marginal means and profile plots depicting these trends confirmed this crossover pattern, where perceived stress increased slightly among intervention participants but decreased among controls from baseline to follow-up. Results of inferential statistical testing showed that there was no significant Time × Group interaction effect, F(1, 19) = 0.15, *p* = 0.701, partial η^2^ = 0.008, indicating that the rate of change in perceived stress did not significantly differ between groups from baseline to follow-up. Additionally, there was no significant main effect of Time, F(1, 19) = 0.00, *p* = 0.968, partial η^2^ < 0.001, indicating that perceived stress did not significantly change from baseline to follow-up when averaging across groups. Finally, there was no significant main effect of Group, F(1, 19) = 0.13, *p* = 0.728, partial η^2^ = 0.007. In sum, perceived stress did not significantly change across time, groups, or the interaction of time and groups.

## 4. Discussion

The goal of this pilot study was to assess the feasibility and short-term efficacy of a 16-week HBST intervention targeting physiological, psychological, and physical health outcomes in obese Latino adolescent males. HBST interventions are much needed, as Latino adolescents experience disproportionately high rates of obesity, stress, and depression, yet typically underutilize existing health resources. In general, the results of this pilot study suggest that the home-based strength training intervention is feasible among obese Latino adolescent males and may affect physiological stress markers. Salivary cortisol was the only dependent variable that showed a statistically significant Time × Group interaction. Changes in muscular strength and anthropometric data were descriptive and exploratory. There were no statistically significant intervention effects on psychological outcomes after controlling for baseline measures. As such, results should be considered exploratory.

### 4.1. Measures of Strength and Anthropometrics

Increases in upper- and lower-body strength were seen in a descriptive manner for the intervention group, while decreases or fewer improvements were seen for control participants. No statistically significant Time × Group interactions were detected for either outcome. While the effect size was moderate for lower-body strength (partial η^2^ = 0.149), the results for this outcome should be interpreted with caution due to the small analytic sample and low power. This effect was consistent with previous studies showing that RT improves muscular strength among adolescents with obesity [18,19,22]; however, due to this being a pilot trial, results should be viewed as exploratory. Future, adequately powered trials should assess whether HBST leads to improvements in muscular strength that are statistically and clinically significant.

Anthropometric outcomes (BMI, BMI percentile, and BMI Z-scores) similarly showed improvements for the intervention group in a descriptive manner without any statistically significant Time × Group interactions. This result is in line with previous work indicating that RT may produce only small improvements in body composition over the short-term when assessed by BMI [5,7]. It is also possible that BMI may not have captured changes in body composition resulting from HBST, as previous studies have shown that increases in lean mass from RT may not correspond to large decreases in BMI [7,17]. While large effect sizes were observed for the Time × Group interaction of BMI Z-scores, this effect was not significant and should be interpreted with caution. Future research should investigate the effects of HBST on these outcomes with larger samples and direct measures of body composition.

### 4.2. Physiological Stress Response

The primary physiological outcome of interest in the current pilot trial was salivary cortisol. Results demonstrated a statistically significant Time × Group interaction (*p* = 0.04). Cortisol decreased among participants randomized to the HBST intervention condition, while cortisol increased among control participants (partial η^2^ = 0.222).

The decrease in cortisol observed among HBST participants when compared to controls may indicate modulation of stress physiology in response to exercise training. Literature in both youth and adults has demonstrated exercise training effects (resistance-based exercise included) on circulating cortisol concentrations and stress physiology more broadly [6,9,10,20]. Physiological measures of stress may also respond to exercise when changes in perceived stress are not observed. Cortisol reactivity has been shown to change in response to exercise training without corresponding improvements in perceived stress [9,14].

Caution should be taken when interpreting these results. In the analytic sample, baseline cortisol concentrations were significantly higher among those randomized to HBST. Regression toward the mean may have played a role in these findings. Additionally, small sample size and high attrition reduce confidence that this effect would remain should the study be replicated with a larger sample. Salivary cortisol was only measured using a single sample at each time point. Cortisol secretion varies by time of day as well as in response to contextual factors. Adherence to the intervention was not objectively monitored and likely varied across participants due to the unsupervised design.

Future studies with sufficient power to detect effects using baseline-adjusted analytic models (i.e., ANCOVA or mixed effects), multiple daily cortisol samples taken across several days, and objective adherence measures are needed to determine if home-based strength training leads to meaningful changes in stress physiology.

### 4.3. Depressive Symptoms and Self-Reported Stress

Results should be interpreted in consideration of group differences at baseline. Participants randomized to the HBST condition reported higher levels of depressive symptoms at baseline compared to controls. While this imbalance could have affected study outcomes by decreasing power to detect a statistically significant Time × Group interaction, descriptive data suggest that HBST did, in fact, lead to improvements in depressive symptoms over time. Because this pilot trial used small samples, this imbalance was likely due to chance rather than randomization failure.

Descriptively, CES-D scores improved over time in the HBST group and got slightly worse among controls. The Time × Group interaction was not significant (*p* = 0.13). Interpretation of these findings is limited by the small analytic sample and imbalance at baseline in depressive symptoms across groups.

Baseline CES-D scores were higher among participants randomized to HBST. Thus, interpretation of the unadjusted improvements in depressive symptoms over time is limited. Regression to the mean may have accounted for some of the observed decrease in depressive symptoms in the HBST group. We performed a sensitivity analysis to further explore this possibility. Results from ANCOVA models with baseline CES-D entered as a covariate revealed that after adjustment for baseline depressive symptoms, differences in CES-D scores across groups at follow-up were not significant. Thus, between-group differences observed when baseline depressive symptoms were not included in statistical models were most likely due to baseline imbalance rather than a true effect of the intervention.

There is some evidence from prior research to support the use of resistance training to reduce depressive symptoms [12,19]. However, results from the current pilot trial do not offer confirmatory evidence that home-based strength training improves depressive symptoms among obese Latino adolescent males. Despite this, the underlying physiological mechanisms that mediate the relationship between exercise and mood are well supported. Improvements in self-efficacy, body image, emotion regulation [12], and neurobiological pathways known to play a role in depression [19] provide rationale for continued exploration of HBST as a treatment modality for depressive symptoms among obese Latino males. Resistance-based exercise may be particularly beneficial to this population given the established gender-related disparities in willingness to seek traditional mental health services among adolescent males [14]. Future studies with adequate power and inclusion of baseline-adjusted analytic strategies are warranted to determine whether HBST produces sustained improvements in depressive symptoms.

In summary, we have shown that HBST is feasible among obese Latino adolescent males and may differentially influence salivary cortisol over time. Although cortisol was the only outcome that demonstrated a statistically significant Time × Group interaction, this result should be interpreted with caution. As previously stated, salivary cortisol was only assessed using a single sample at each time point. Diurnal variation was not considered, nor were participants controlled for sleep, dietary intake, and psychosocial stress exposure. Thus, while these data are promising, conclusions regarding the impact of HBST on stress-related physiological pathways cannot be made. Future studies should include larger sample sizes, repeated measures of cortisol throughout the day, and control for important confounders to determine whether HBST has the ability to produce sustained and clinically significant changes in physiological stress regulation.

Levels of perceived stress, as assessed by the PSS-14, were not statistically significantly different across time for either group. Prior research has also demonstrated null effects of acute stress management interventions on perceived stress [9,24]. However, perceived stress among immigrant adolescents may be a result of chronic contextual stressors that were not influenced by participation in the current study, such as acculturative stress.

### 4.4. Feasibility

As obesity prevention continues to gain attention, researchers should also consider developing and testing interventions that reduce obesity risk and promote health in adolescent populations. Recruitment into research studies is one way to identify and reach youth populations to begin behavioral interventions at an early age. A secondary aim of the current pilot study was to assess the feasibility of delivering an HBST intervention to obese Latino adolescent males. Obese Latino youth may be less likely to participate in clinic- or gym-based exercise programs due to financial burdens, transportation limitations, lack of time, and/or unstructured facility access [8,15]. HBST interventions serve as an opportunity to provide adolescents the structure and tools needed to engage in exercise behavior outside of clinical or school environments.

Results from this pilot trial suggested mixed levels of feasibility for the intervention. On one hand, we were able to meet our recruitment target, which suggests initial interest and potential acceptability of strength-based interventions within Latino adolescent males. However, retention was low within both conditions and even lower within the HBST group. Maintaining adolescents’ participation in a 16-week unsupervised HBST program may be difficult without periodic face-to-face contact. Given the age of the sample, it is possible that school obligations, family duties, or other after-school activities may have taken priority over study activities, leading participants to drop out of the study. Despite our efforts to conduct exploratory analyses to determine if there were systematic differences at baseline between those who completed versus did not complete the study (SES, BMI z-score, age, ethnicity), our small sample precluded any definitive conclusions.

Due to the importance of retention when examining intervention feasibility, strategies to improve retention should be considered for future studies. Ideas may include methods of accountability, such as providing participants with scheduled time points to check in, utilizing text message or app-based reminders, web-based interactive programs, wearable activity trackers, or regular phone contact. Using some of these methods may provide a more supportive framework for future home-based behavioral interventions.

### 4.5. Limitations

Several limitations of this pilot trial should be considered. First, a small sample size with high attrition limited power and may have masked a treatment effect. Although we suspect differential retention between groups biased our results, retention and adherence are commonly known limitations to behavioral interventions among adolescents and other hard-to-reach populations. Second, we cannot make definitive conclusions that differences in CES-D scores were produced by our intervention given group differences at baseline in depressive symptom severity. Although our groups were randomized, sometimes random chance produces group differences at baseline (which are more likely to happen in small pilot studies like this) that can mask true treatment effects. Third, our analytic strategy was limited by our sample size. Although we opted to use repeated-measures ANOVA because we had two fixed timepoints and this was a pilot study looking at differences between two conditions (making ANOVA a suitable analysis strategy), we would utilize a more flexible analytic approach that could accommodate our study design (linear mixed-effects modeling) if we had a larger sample. Group differences at baseline in depressive symptoms and cortisol in our final analytic sample could have impacted our ability to draw comparisons between groups. Although participants were appropriately randomized to conditions, we suspect that differential attrition played a role. Baseline differences in cortisol and depressive symptoms observed in the analytic sample likely reflect chance variation and differential attrition in this small pilot trial rather than systematic allocation bias. These imbalances were considered when interpreting between-group changes. Future, well-powered trials using analytic techniques that account for baseline values (i.e., ANCOVA or mixed effects modeling) would be beneficial. Fourth, because the intervention was home-based and unsupervised, we were unable to track objective adherence/exercise fidelity. Adherence was determined by self-reported training logs, which are susceptible to reporting bias. We recommend that future studies embed objective monitors (e.g., wearable activity trackers, app-based logging systems, and remote monitoring platforms) to ensure intervention fidelity and improve adherence assessments. Adherence to the intervention may have also driven heterogeneity in our outcomes. Additionally, because motivational interviewing was delivered in conjunction with strength training, we cannot make claims that HBST worked to produce our observed effects. Fifth, we only collected one sample of salivary cortisol at each time point. As such, we cannot make any claims that chronic dysregulation of the HPA axis was present. Lastly, depressive symptoms and perceived stress were both self-reported and may be impacted by the participant’s willingness to report these variables. Mental health was only assessed at baseline, 3 months, and 6 months. It is possible participants experienced periods of worsening or improving self-reported mental health that we were unable to detect due to limited assessment time points. Future RCTs should assess cortisol using multiple days of sampling and utilize more frequent assessments of mental health as well as objective measures to track adherence (wearable technology).

### 4.6. Strengths

There are several strengths of this pilot study. First, the present study fills an important gap in the literature by targeting obese Latino adolescent males. Latino adolescents experience disproportionate rates of obesity, stress, and depressive symptoms and represent an understudied sample in intervention research examining these conditions. Addressing this disparity is critical to understanding how to best make health interventions accessible to this growing population.

Second, due to the home-based nature of the strength training intervention used in the current study, it can be both easily implemented and low in cost. The unsupervised nature of this intervention may also eliminate common barriers experienced when enrolling participants into an exercise program (i.e., transportation issues, time, lack of facilities). Third, the current study uses both self-report (CES-D, PSS-14) and biological outcomes (salivary cortisol). This allows for an objective biomarker of stress to complement the self-reported stress scores and bolsters conclusions made about potential mechanisms of change for mental health outcomes.

Finally, the randomized controlled pilot design and utilization of standardized valid/credible measurement tools allow for confidence in the internal validity of the results. Though this study is limited by its pilot nature, results can be used to inform future powered trials. Implementing HBST interventions may be one strategy to target physiological and mental health outcomes in Latino adolescents. The benefits of HBST on stress-related biological pathways can play an important role in obesity prevention. If HBST can improve strength and other health markers without participants having to visit a gym or purchase expensive equipment, HBST could significantly decrease obesity rates. Furthermore, motivational interviewing used in this intervention has also been shown to promote health behavior change.

## 5. Conclusions

In conclusion, this pilot study suggests that an HBST intervention is feasible among obese Latino adolescent males and may influence physiological stress measures differentially over time. While most outcomes were not statistically significant, improvements in strength and body composition were seen descriptively. Only salivary cortisol significantly differed between conditions at follow-up (i.e., demonstrated a Time × Group interaction), but this result should be interpreted with caution given baseline imbalance, the small sample size, and study limitations (single time-point cortisol sampling without accounting for diurnal rhythm or possible confounders). Strengths of the current study include its novelty as one of the first RCTs to explore the efficacy of an HBST program among Latino adolescents. Due to the exploratory nature of this secondary pilot analysis, definitive conclusions cannot be made about intervention effects. Future studies with appropriate power and improvements such as baseline-adjusted analytic methods, repeated physiological measures, extended follow-up assessments, and objective adherence checks are warranted to assess whether HBST results in meaningful changes in physiological or psychological factors.

The generalizability of our results is limited. First, our sample only included obese Latino adolescent males. Second, participants were recruited from one geographical area. Finally, attrition limited our final analytic sample. Therefore, our results may not generalize to adolescent females, males of other races/ethnicities, adolescents without obesity, or individuals who live in different sociocultural or geographical regions.

## Figures and Tables

**Table 1:** Baseline Characteristics of Evaluable Participants (N = 25).

	Control (N = 14)	HBST (N = 11)
Age (Years)	15.75 ± 0.71	15.29 ± 1.07
Height (M)	1.65 ± 0.22	1. 63± 0.36
Weight (kg)	94.07 ± 19.93	101.6 ± 18.20
BMI (kg/m^2^)	32.06 ± 3.08	32.83 ± 4.57
BMI Percentile	98.20 ± 1.72	98.10 ± 1.56
Z-Score	2.21 ± 0.32	2.23 ± 0.41
CES-D Score	13.50 ± 3.98	20.75 ± 9.15
PSS Score	21.38 ± 2.99	20.38 ± 1.60
Cortisol (pg/mL)	9.99 ± 3.47	14.93 ± 4.28

Note. Values are presented as mean ± standard deviation. CES-D = Center for Epidemiologic Studies Depression Scale; PSS = Perceived Stress Scale.

**Table 2:** Strength: Across Groups Effects for Evaluable Participants (N = 25).

	Control (N = 14)		HBST (N = 11)	
	Baseline	Follow-up	Baseline	Follow-up
Arm 1RM (lbs)	181.79 ± 58.56	173.57 ± 75.94	184.38 ± 60.68	200.00 ± 71.91
Leg 1RM (lbs)	522.50 ± 144.92	454.29 ± 146.81	491.11 ± 167.07	587.22 ± 264.61

Note. 1RM = one-repetition maximum.

## Data Availability

Due to ethical and IRB restrictions and the presence of sensitive human participant data, the dataset is not publicly available. The dataset cannot be made publicly available. Upon request, and subject to institutional approval and data use agreement, the authors may be able to provide access to the de-identified data. Please contact the corresponding author with reasonable requests.
